# High Mortality Rate due to False Gid in a Sheep Herd

**DOI:** 10.1155/2013/650358

**Published:** 2013-08-12

**Authors:** Ali Asghar Mozaffari, Saeedeh Shojaeepour, Shahin Ghahremani Ghareh Cheshmeh

**Affiliations:** ^1^Department of Clinical Studies, School of Veterinary Medicine, Shahid Bahonar University of Kerman, P.O. Box 76169 14111, Kerman, Iran; ^2^Education and Research Hospital, School of Veterinary Medicine, Shahid Bahonar University of Kerman, P.O. Box 76169 14111, Kerman, Iran

## Abstract

The sheep nasal bot, *Oestrus ovis* (Diptera: Oestridae), is a cosmopolitan parasite commonly found in sheep and occasionally goats. Rarely a bot will migrate into the sheep brain (false gid). Following the complaint of an animal husbandman about high mortality rate in a sheep herd, the herd was clinically, hematologically, and pathologically examined exactly. Clinical, hematological, and pathological findings were described in the text. Necropsy findings showed heavy infestation with *Oestrus ovis* larvae. The herd was treated with Ivermectin. After treatment all patients without nervous sings were recovered. Patients with nervous signs did not respond to treatment, but new cases of disease did not occur and the mortality stopped. In the present report, a high mortality rate due to false gid in a sheep herd was described. The prevention and control of the disease are important because of economic losses and the possibility of transmission to the human.

## 1. Introduction

The sheep nasal bot, *Oestrus ovis *(Diptera: Oestridae), is a cosmopolitan parasite commonly found in sheep and occasionally goats. The disease has a higher prevalence in tropical areas. Adult flies deposit larvae in or near the sheep's nostrils and the first instars move into the nasal passages where they feed on nasal secretion. Larvae migrate to the frontal sinuses and complete two molts before returning to the nasal passages, from where they are expelled by sneezing [1, 2]. The duration of this parasitic portion of the life cycle varies considerably from a few weeks to several months, depending on the season and climatic conditions [3]. Clinical symptoms, depending on the infected area and larvae numbers, are different. Clinical respiratory signs such as seromucous or purulent nasal discharge, frequent sneezing, and dyspnea may severely impair the health of affected animals [4]. Rarely a bot will migrate into the sheep brain (false gid) [5]. In the present report, a high mortality rate due to false gid in a sheep herd was described.

## 2. History and Results

**Figure 1:**
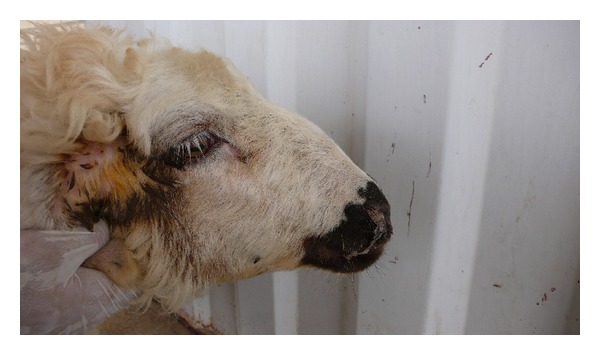
Serosanguinous nasal discharge in a sheep with false gid.

**Figure 2:**
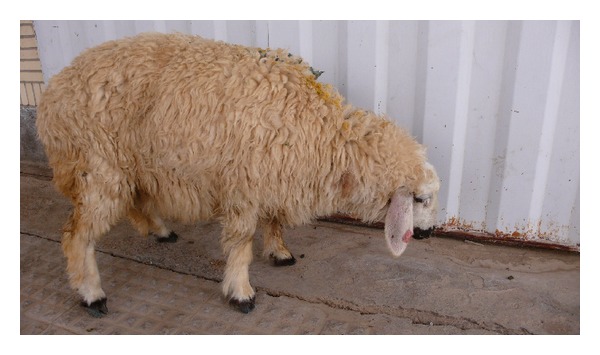
Depression in a sheep with false gid.

**Figure 3:**
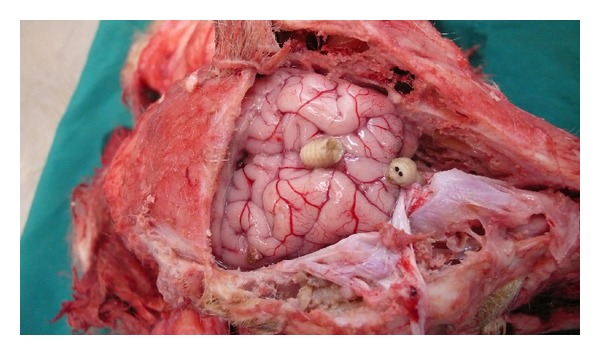
The larvae in nasal passage and brain of a sheep with false gid.

Following the complaint of an animal husbandman about high mortality rate (10%) in a sheep herd, the herd was clinically, hematologically, and pathologically examined exactly. In clinical examination, clinical signs such as sneezing, serosanguinous nasal discharge (Figure 1), hyperemic nasal mucosa, depression (Figure 2), circling, head pressing, aimless wandering, compulsive walking, blindness, and ataxia were observed. Vital signs including temperature, heart, and respiratory rates were normal. Hematological examination including RBC, PCV, and total and differentiated WBC was also in normal range. In necropsy, the larvae were found in nasal passage, frontal sinuses, and brain (Figure 3) and the nasal mucosa was hyperemic. The herd was treated with Ivermectin 1% (Ivectin, Razak Co., Tehran, Iran) at dose rate of 0.2 mg/kg bodyweight. After treatment all patients without nervous sings were recovered. Patients with nervous signs did not respond to treatment, but new cases of disease did not occur and the mortality stopped. 

## 3. Discussion 

*Oestrus ovis* is a problem of sheep and goat worldwide, as the larvae cause a serious mucopurulent myiasis of the nasal passages and frontal sinuses. There may be also erosion of the bones of the skull and damage to the brain, leading to blind staggers or false gid [6, 7]. The migratory larvae penetrate and erode the dorsal turbinate bones, frontal sinuses, and occasionally the skull bones while entering into the cerebral cavity, causing false gid [8]. Heavy infection can induce a condition known as false gid in which the affected animal becomes unthrifty and exhibits a lack of coordination and staggers around in circles [5]. The prevalence of *O. ovis* infestations in sheep in the world is as follows: 33.2–65% in France, 71.1% in Spain, 55.8–91.0% in Italy, 22.6% in Libya, 17.2% in Iraq, 8.7% in Egypt, 58% in Jordan, 5.5% in Saudi Arabia, 21.0% in Ethiopia, 10–100% in Morocco, 67.4% in Algeria, 6–52% in Zimbabwe, and 8.1% in India [6, 9–21]. There is no report about false gid in the word up to now. In the present report, a high mortality rate due to false gid in a sheep herd was described. The prevention and control of the disease are important because of economic losses and the possibility of transmission to the human [1].
